# Fractal analysis: a new biomarker for determining clot characteristics in critically ill patients

**DOI:** 10.1186/cc11037

**Published:** 2012-03-20

**Authors:** GR Davies, SN Stanford, MJ Lawrence, D Gill, PR Williams, K Morris, D Thomas, PA Evans

**Affiliations:** 1 NISCHR Haemostasis Biomarker Research Unit, Swansea, UK

## Introduction

Recent research [1] has highlighted a novel new biomarker of haemostasis: the fractal dimension (Df). This new biomarker relates the kinetics of clot formation to clot outcome in whole blood and allows us to quantify the complexity of the fibrin network microstructure which is believed to be the template for development of the mature clot. It is well established that abnormalities in haemostasis contribute to the pathogenicity of critical illness [2]. This prospective study aims to assess the effect of critical illness on clot structure and monitor the sensitivity of Df to therapeutic intervention.

## Methods

Patients with critical illness inducing SIRS were recruited on admission to the intensive therapy unit in a large teaching hospital in Wales. Blood was taken for routine coagulation testing, ROTEM thromboelastometry and rheological analysis (Df and Tgel) on admission, at 6 hours and 24 hours to assess pathophysiological state and progression. Twelve patients were recruited: nine severe sepsis and three severe DKA with metabolic disorder. Twelve healthy volunteers were recruited as a matched control.

## Results

**Figure 1:**
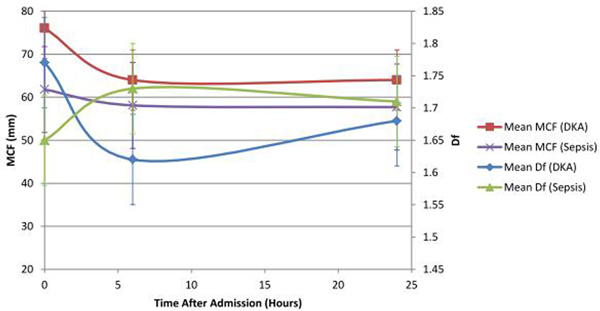


Mean Df in the control group was 1.73 ± 0.03 whereas mean Df in DKA and sepsis was found to be 1.77 ± 0.07 and 1.65 ± 0.05 respectively. Marked differences were observed in Df and maximum clot firmness (MCF) in response to treatment intervention (Figure 1). Furthermore, patients saw a dramatic decrease in Df post enoxaparin, but no significant change in MCF was observed (Table 1).

**Table 1:** 

	Mean Df	Mean MCF (mm)
Pre enoxaparin	1.79 ± 0.08	68.0 ± 8.0
Post enoxaparin	1.64 ± 0.10	64.3 ± 4.2

## Conclusion

Df shows specificity between severe DKA and sepsis. Df shows sensitivity to treatment intervention and illness progression in the critically ill.
